# Artificial Intelligence and Digital Technologies in *Prakriti* Assessment: Toward Standardized Evidence-Based Ayurvedic Practice

**DOI:** 10.1016/j.jaim.2026.101372

**Published:** 2026-08-03

**Authors:** Ramakrishna Allam, B. Kothainayagi, Sankari Subbiah, Swagata Sarkar, Sivgaamasundari R

**Affiliations:** 1 Sri Sri College of Ayurvedic Science and Research Hospital, Sri Sri University, Cuttack, Odisha. India; 2 The T.N. Dr. M.G.R Medical University, Chennai, Tamilnadu. India; 3 Sri Sai Ram Engineering College, Chennai, India; 4 Sri Sairam Ayurveda Medical College and Research Centre, The T.N. Dr. M.G.R Medical University, Chennai, Tamilnadu. India

**Keywords:** Ayurveda, Artificial intelligence, Body constitution, Machine learning, *Prakriti*, Sensors

## Abstract

Prakriti, the Ayurvedic concept of individual somatic constitution, forms the foundation for personalized preventive and therapeutic strategies by classifying individuals based on distinctive physical, physiological, and psychological attributes. Traditional methods of *Prakriti* assessment, such as physician-administered questionnaires, clinical examinations, and observational techniques, though deeply rooted in Ayurvedic principles, are often constrained by subjectivity, limited reproducibility, and a lack of standardization.

Recent advancements in Artificial Intelligence (AI), Machine Learning (ML), and digital health technologies offer new opportunities to modernize *Prakriti* assessment and enhance its reliability. This narrative review systematically located, selected, and synthesized relevant literature from scientific databases and institutional repositories to identify digital, AI, and ML tools applicable to the parameters outlined in the Central Council for Research in Ayurvedic Sciences (CCRAS) *Prakriti* Assessment Manual. Retrieved data were categorized according to their relevance to physical, physiological, psychological, and behavioral domains, emphasizing technologies that enable objective measurement and computational analysis.

The review highlights the potential of integrating physiological sensors, image analysis systems, and computational algorithms with classical Ayurvedic approaches to improve the accuracy and clinical relevance of *Prakriti* analysis. The convergence of Ayurveda and digital health technologies holds transformative potential to generate evidence, enhance clinical applicability, and foster interdisciplinary research in personalized medicine. However, challenges related to data quality, interoperability, algorithmic interpretability, and domain-specific validation must be addressed to ensure credibility and wider adoption. Overall, digital innovations can significantly enhance the rigor, reach, and impact of *Prakriti* assessment in integrative healthcare.

## 1 Introduction

Ayurveda, an ancient holistic health system, prioritizes individualized care through the principle of *Prakriti*, the intrinsic somatic constitution formed at conception and regarded as stable throughout life [1]. *Prakriti*, captures the dynamic interplay and dominance of the three doshas: *Vata* (associated with movement and cognitive processes), *Pitta* (linked to metabolism and thermoregulation), and *Kapha* (responsible for cohesion and fluid balance), shaping an individual’s physical profile, physiological responses, temperament, and behaviors [2], [3], [4]. Precise *Prakriti* evaluation is foundational for Ayurvedic diagnostics and formulation of interventions, informing recommendations on lifestyle, nutrition, and treatment pathways [5].

Historically, assessment of *Prakriti* has relied on trained practitioners who conduct detailed clinical observations, structured interviews, and utilize traditional methods such as *Nadi Pariksa* (pulse examination) [6], [7]. While these practices uphold classical Ayurvedic traditions, they introduce subjectivity and variability, posing challenges to reproducibility and limiting standardization across diverse clinical settings [8]. The rapid advancement of Artificial Intelligence (AI) and Machine Learning (ML) is transforming healthcare, enabling objective analytics, individualized treatment, and intelligent diagnostic tools worldwide [9].

In this context, leveraging digital innovations, including wearable sensors, smartphone platforms, automated facial and speech analysis, and AI-based classifiers, presents an avenue to enhance accuracy and reproducibility in *Prakriti* categorization [10]. This narrative review explores the intersection of Ayurvedic doctrine and digital technologies in modern *Prakriti* assessment, synthesizing available research, established capabilities, barriers to implementation, and prospects for future development. Aligning age-old Ayurvedic wisdom with contemporary computational strategies supports the advancement of integrative healthcare that is both scientifically rigorous and true to its classical roots.

## 2 Methodology

To systematically find, choose, extract, and synthesize information on the integration of digital technologies, ML, and AI in Ayurvedic *Prakriti* evaluation, this study used a structured narrative review technique. The goal was to provide a standardized, evidence-based paradigm for *Prakriti* evaluation by combining transdisciplinary insights from Ayurveda, computer sciences, and biomedical engineering.

### 2.1 Locating data

A comprehensive literature search was conducted across PubMed/MEDLINE, Scopus, Web of Science, and Google Scholar from database inception to January 2026. Additional sources including the Ministry of AYUSH, the World Health Organization (WHO), IEEE Xplore, and reputable biomedical technology repositories were also investigated to guarantee the inclusion of gray and technical literature.

The search combined controlled vocabulary (MeSH) and free-text keywords, organized into three conceptual domains:

1. Ayurveda and Prakriti (*Ayurveda, Dosha, Prakriti, constitutional typing*);
2. Artificial Intelligence and Digital Technologies (*AI, ML, deep learning, sensors, image processing, digital health*); and
3. Assessment and Standardization (*diagnosis, classification, validation, personalized medicine*).

Boolean operators (AND/OR) and truncations were applied. The search was limited to publications in English, Hindi, and Sanskrit, with automatic alerts set to capture updates during the review period

### 2.2 Selecting Data

The selection process adhered to predefined inclusion and exclusion criteria.

Inclusion criteria:

• Peer-reviewed articles or review papers discussing the application of AI, ML, or digital technologies in *Prakriti* assessment.
• Studies employing computational, sensor-based, or image-based tools for the evaluation of Ayurvedic parameters.
• Studies referencing or aligning with Central Council for Research in Ayurvedic Sciences (CCRAS)-based diagnostic parameters
• Research consistent with diagnostic frameworks recommended by the CCRAS or equivalent guidelines.

Exclusion criteria:

• Non-peer-reviewed sources, duplicates, and redundant conference abstracts.
• Publications lacking empirical, computational, or clinical relevance to *Prakriti* assessment.
• Studies limited to theoretical Ayurvedic concepts without digital or analytical components.

### 2.3 Extracting data

For each eligible study, data were extracted using a structured template capturing: study design, sample characteristics, type of digital (wearable sensors, imaging systems, Natural Language Processing (NLP) tools) or AI/ML tools, validation methods, and observed outcomes. Studies incorporating multimodal tools, such as image, sensor, and questionnaire-based data, were grouped accordingly.

### 2.4 Synthesizing data

Extracted data were narratively synthesized to highlight digital and computational innovations that improve objectivity, standardization, and reproducibility in *Prakriti* assessment. Findings were categorized across four domains, physical, physiological, psychological, and behavioral, demonstrating how AI and ML tools enable data-driven interpretation consistent with the CCRAS questionnaire. Comparative analysis was further conducted between traditional assessment techniques and AI-based models to delineate improvements in objectivity, scalability, and clinical reproducibility.

**Figure 1:**
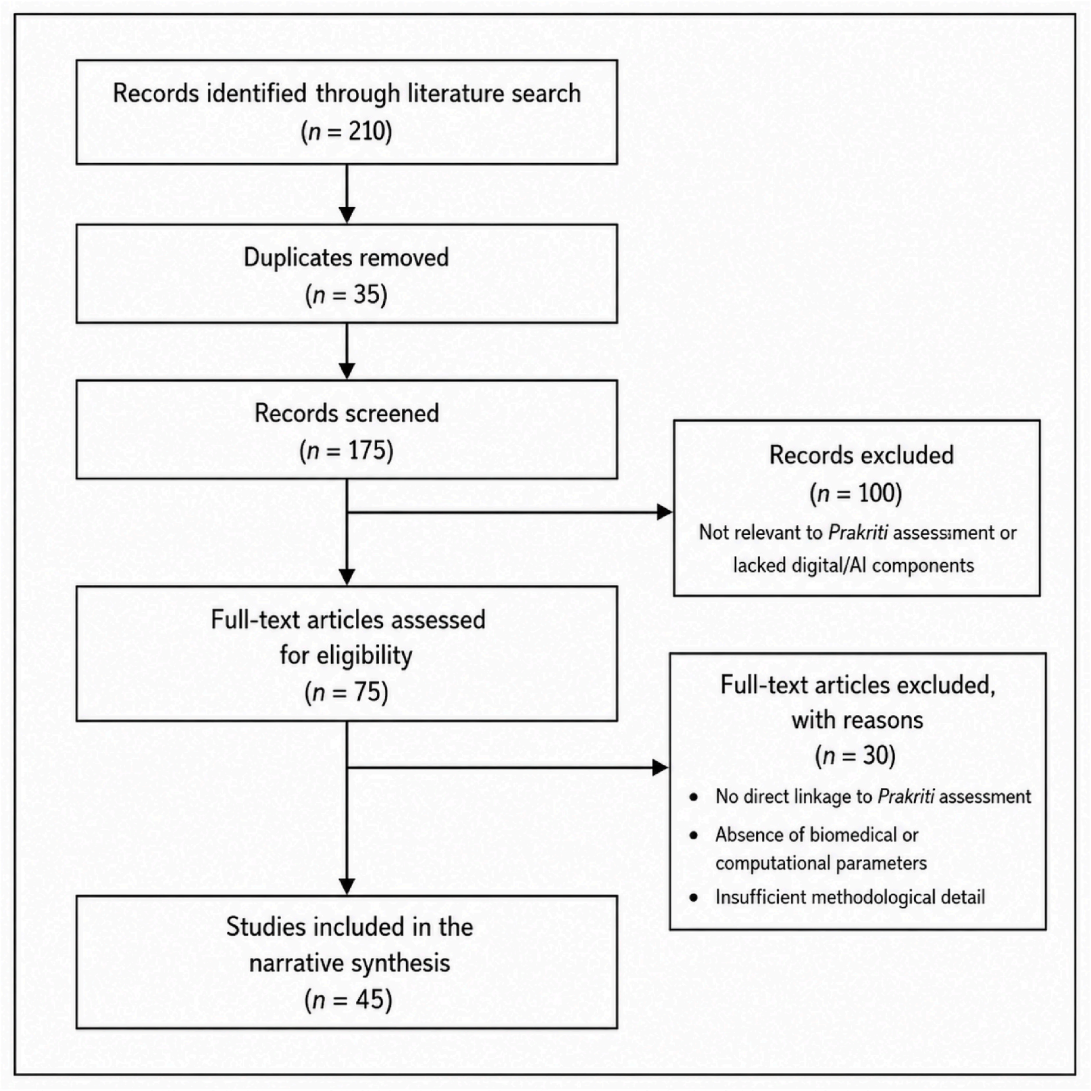
PRISMA flow diagram of study selection process

A comprehensive literature search yielded a total of 210 records. After removal of duplicates (n = 35), 175 records were screened based on title and abstract. Of these, 100 records were excluded due to lack of relevance to Prakriti assessment or absence of digital/AI components. 75 full-text articles were assessed for eligibility, and 30 articles were excluded for reasons including, absence of diagnostic linkage to *Prakriti* or biomedical parameters, lack of computational methodology, or insufficient methodological detail. Finally, 45 studies were included in the narrative synthesis (Fig. 1). Although this study was designed as a narrative review, the selection and screening process broadly followed PRISMA principles to enhance transparency and reproducibility.

### 2.5 Parameters for *Prakriti* assessment

This narrative review focuses on the use of digital technologies and AI to assess various physical, physiological, psychological, and behavioral traits essential for *Prakriti* evaluation, as detailed in Table 1. These parameters are based on the standardized *Prakriti* assessment framework developed by the CCRAS, which serves as the foundation for individualized Ayurvedic health interventions [11].

**Table 1:** Parameters of *Prakriti* analysis

Parameter Category	Parameters
Physical	Age, Weight, Height, BMI, Skin texture and color, Hair color and texture, Teeth color and arrangement, Eye size and color, Anthropometric measurements (waist-hip ratio, mid-arm circumference, limb proportions), Nail color, size and texture, Forehead size, Body musculature, Palm and lip color and texture,
Physiological	Gait and body movements, Voice strength and speech pattern, Appetite, Thirst, Sweating, Bowel habits, SPO2, Respiration rate, Heart rate, Pulse, ECG, Nadi (pulse diagnosis)
Psychological	Intelligence, Memory, Decision-making ability, Emotional stability
Behavioral	Friendship tendencies, Way of talking

## 3 Limitations of current *Prakriti* evaluation methods and need for standardization

### 3.1 Subjectivity and low inter-rater reliability

A major limitation of *Prakriti* assessment is its reliance on subjective judgment by Ayurvedic practitioners. Traditional evaluations are based on visual inspection, patient interviews, and diagnostic methods such as *Nadi Pariksa* (pulse examination). These approaches depend heavily on practitioner expertise and intuition, which often results in variability. Reported inter-rater reliability values are generally low, ranging between 0.20 and 0.40 even among experienced physicians, highlighting the inconsistency in classification [12], [13], [14], [15].

### 3.2 Absence of standardized and validated tools

Currently, no universally accepted or psychometrically validated questionnaire exists for *Prakriti* determination. Multiple institutions and researchers have developed their own tools, but they vary considerably in structure, dosha scoring systems, and interpretation methods. This lack of uniformity makes comparison across studies difficult. Furthermore, essential psychometric properties, such as construct validity, test-retest reliability, and internal consistency, have not been comprehensively established for most available instruments [8].

### 3.3 Qualitative nature of assessment parameters

Key indicators of *Prakriti*, including skin texture, digestion quality, mental disposition, and behavioral patterns, are primarily qualitative. The absence of precise, quantifiable measures limits opportunities for digitization and statistical modeling. As a result, reproducibility, scalability, and objective validation remain significant challenges in both clinical practice and research [8].

### 3.4 Limited adoption of digital and health informatics tools

Digital technologies, such as mobile health (mHealth) platforms, wearable biosensors, and AI-driven diagnostic systems, have seen minimal application in *Prakriti* analysis. Most assessments are still carried out manually, with limited use of electronic health records or structured digital databases. This restricts the generation of large-scale datasets required for machine learning, population-level analyses, and cross-cultural validation.

### 3.5 Scarcity of open datasets and interdisciplinary collaboration

Another pressing issue is the lack of publicly available, well-labeled datasets combining *Prakriti* classifications with clinical, physiological, or image-based parameters. The absence of such resources hampers the training and validation of machine learning algorithms. In addition, insufficient collaboration between Ayurvedic experts and specialists in fields such as data science, biomedical engineering, and psychology has slowed innovation in developing objective and evidence-based assessment models**.**

To modernize *Prakriti* assessment and align it with global standards of evidence-based healthcare, foundational efforts are required in three key areas: (i) development of validated and standardized tools, (ii) creation of open-access, multimodal datasets, and (iii) fostering interdisciplinary research collaborations. Such measures will enable the establishment of a reliable, scalable, and globally relevant framework for *Prakriti* assessment that integrates classical Ayurvedic wisdom with modern scientific methodologies.

## 4 Reimagining *Prakriti* analysis through AI and emerging technologies

Traditional *Prakriti* assessment in Ayurveda relies on clinical observation and physician expertise, making it subjective and inconsistent. Advances in digital health and AI now enable objective and reproducible models by integrating diverse data sources such as biometric signals, morphological traits, and psychological markers.

Tools like wearable sensors, image and speech analysis, mHealth platforms, and machine learning allow a multidimensional approach that enhances diagnostic accuracy, reduces observer bias, and generates standardized datasets for clinical and research use. AI frameworks also support validation against classical questionnaires, improve inter-rater reliability, and strengthen evidence-based integrative practice. Merging Ayurvedic principles with computational intelligence thus creates a foundation for modern, personalized healthcare.

### 4.1 Key technologies and their AI integration

The following subsections highlight specific technological domains and their integration with AI in advancing *Prakriti* assessment.

#### 4.1.1 Physical parameters

Recent advances in AI and ML provide powerful tools to enhance *Prakriti* assessment by objectively analyzing a wide range of physical traits. ML models can process biometric data from smart devices to calculate weight, height, and BMI [16], while computer vision algorithms evaluate skin texture, including pore size, smoothness, wrinkles, and pigmentation, to identify characteristics corresponding to Ayurvedic skin types [17], [18], [19], [20]. Similarly, AI-based image analysis can classify hair texture and color, detecting features such as dryness, oiliness, or graying patterns, which can inform dosha-related profiling [20], [21], [22]. Dental and ocular features can also be assessed: 3D intraoral scanning combined with ML can analyze tooth alignment, spacing, and color [23], [24], while high-resolution eye imaging allows evaluation of iris color, scleral health, and morphology, offering insights into constitutional traits [25], [26], [27].

AI-driven body scanning technologies and electromyography (EMG) provide precise anthropometric measurements, including limb lengths, torso proportions, and musculature distribution [28], [29], [30], enabling classification into *Vata*, *Pitta*, or *Kapha* types. High-resolution imaging of nails [31], forehead [32], palms, lips, and facial geometry further supports assessment of texture, color, and structural variations relevant to Ayurvedic diagnostics [20], [33], [34], [35], [36] (Table 2).

**Table 2:** Advanced technologies for physical parameters in *Prakriti* assessment

Parameters	Technology used
Age	Digital questionnaires can be used to estimate age.
Weight, height, and BMI	Sensor-based scales, wearable devices, and cameras
Skin Texture and Color	Spectrophotometers, cameras with high resolution, multispectral imaging, and computer vision systems
Hair color and texture	AI-driven dermatology tools, digital imaging, and 3D scanners.
Teeth color and arrangement	3D intraoral scanners, dental imaging, and computer vision technologies.
Eye size and color	Ophthalmic scanners, digital imaging, and facial recognition technologies.
Anthropometric measurements	3D body scanners, laser-based measurement tools, and motion capture technologies.
Nail color, size and texture	Digital imaging, spectral sensors, and macro cameras.
Forehead size	Facial recognition systems, 3D imaging, and computer vision algorithms.
Body musculature	3D body scanners and electromyography (EMG) devices.
Palm, lip color, and texture	Digital imaging, 3D scanners, and high-resolution cameras

#### 4.1.2 Physiological parameters

AI and ML are increasingly applied to assess dynamic physiological traits relevant to *Prakriti* evaluation. Wearable devices equipped with accelerometers and gyroscopes capture gait and body movement in real time diagnostics [37], allowing ML algorithms to analyze balance, coordination, and walking patterns [38], which Ayurveda associates with *dosha* characteristics. Voice analysis tools can evaluate speech strength, tone, pitch, and rhythm [39], enabling ML models to detect traits such as fast or slow speech and tonal variations, which correspond to *Vata*, *Pitta*, or *Kapha* tendencies. Biosensors integrated into wearable devices monitor parameters such as hydration, perspiration, hunger, and thirst [40], [41]; ML algorithms interpret deviations from normative ranges to identify potential doshic pattern recognition.

AI-enabled smart toilets and gastrointestinal sensors track bowel movement frequency and consistency [42], [43], providing objective data on digestive patterns linked to *Prakriti*. Similarly, wearable devices such as smartwatches monitor vital signs including heart rate, oxygen saturation, and respiration rate [44], while ECG analysis allows AI models to detect irregularities and infer dosha-related trends. Pulse diagnosis, a cornerstone of Ayurvedic evaluation, can be digitized using pressure sensors and wearable pulse monitors; AI algorithms analyze variations in rhythm, strength, and frequency to classify *Vata*, *Pitta*, or *Kapha* types [45], [46]. Sleep and cognitive patterns are assessed via EEG headbands and sleep trackers [47], [48], [49], [50], [51], with AI analyzing brainwaves, sleep stages, and heart rhythms to identify deviations indicative of doshic pattern recognition (Table 3). Collectively, these technologies offer a quantitative, reproducible, and integrative framework for modernizing *Prakriti* assessment while preserving Ayurvedic principles.

**Table 3:** Advanced technologies for physiological parameters in *Prakriti* assessment

Parameters	Technology used
Gait and body movement	Wearable sensors, motion capture systems, accelerometers, gyroscopes, and video-based analysis.
Voice strength and speech pattern	Microphones, voice recognition software, and NLP (Natural Language Processing) algorithms.
Appetite, thirst, and sweat	By digital questionnaire Wearable biosensors, hydration tracking devices, and smart clothing.
Bowel habits	By digital questionnaire Smart toilets, gastrointestinal trackers, and wearable devices for gut monitoring.
SPO2, respiration rate, heart rate, and pulse ECG	Pulse oximeters, wearable fitness trackers, and smartwatches.
Nadi (Pulse diagnosis)	Pressure sensors, wearable pulse monitors, and digital Nadi sensors.
Sleep patterns, brain activity	Wearable sleep trackers, polysomnography systems, electrocardiogram (ECG) devices, and electroencephalogram (EEG) sensors.

#### 4.1.3 Psychological parameters

AI and ML are increasingly applied to evaluate cognitive and psychological traits relevant to *Prakriti* assessment. Intelligence and memory can be assessed using AI-based cognitive tests and neuroimaging tools, which analyze brain activity and performance in tasks measuring attention, memory recall, problem-solving, and learning speed. ML algorithms trained on large datasets can identify patterns in cognitive performance, predict potential cognitive decline, or suggest targeted interventions. In Ayurvedic terms, these measures can help detect dominance of the *Sattva* (calmness), *Rajas* (agitation), or *Tamas* (lethargy), reflecting an individual’s mental and emotional constitution. Tools such as Lumosity, CogniFit, and EEG-based devices like Muse provide real-time monitoring and analysis that can be adapted for *Prakriti* evaluation [51], [52], [53], [54].

Decision-making ability, another key psychological trait, can be quantified through AI systems that simulate complex, time-sensitive, or emotionally charged scenarios. ML models track response times, choice complexity, risk assessment tendencies, and cognitive flexibility, offering insights into mental clarity and focus [55]. For example, dominant *Vata* individuals may demonstrate impulsive decisions, while *Kapha* types tend to show slower but more stable responses. Emotional stability can also be monitored via AI tools analyzing facial expressions, voice tone, and physiological signals such as heart rate variability and galvanic skin response [56], [57]. Platforms like Affectiva and Cogito utilize these measures to provide real-time emotional profiling, enabling assessment of *Sattva* (calmness), *Rajas* (agitation), or *Tamas* (lethargy), thereby linking cognitive and emotional health with Ayurvedic constitution (Table 4).

**Table 4:** Advanced technologies for psychological parameters in *Prakriti* assessment

Parameters	Technology used
Intelligence and memory	Cognitive assessment platforms, brain-computer interfaces (BCIs), neuroimaging tools (e.g., fMRI, EEG), and mobile-based cognitive apps.
Decision-Making power	Decision-support systems, behavioral analysis platforms, machine learning models for decision-tree analysis, and AI-enabled questionnaires.
Emotional stability	Facial emotion recognition software, sentiment analysis, wearable devices for physiological monitoring (e.g., heart rate variability), and behavioral tracking apps.

#### 4.1.4 Behavioral parameters

AI and ML can also be leveraged to evaluate social and behavioral characteristics relevant to *Prakriti*. Friendship tendencies, which reflect how individuals form and maintain social bonds, can provide insights into personality traits associated with different *doshas*. AI algorithms analyze social media activity, messaging patterns, and interaction frequency to identify behavioral tendencies.

NLP refers to a class of computational techniques that enable computers to process, interpret, and generate human language (both text and speech) using methods from AI, ML, and computational linguistics. Common NLP frameworks include Python-based libraries such as NLTK, spaCy, TensorFlow, PyTorch, and transformer-based architectures, which may be adapted for analyzing speech patterns, sentiment, and behavioral traits relevant to *Prakriti* assessment. NLP models evaluate textual conversations for friendliness, openness, and emotional intelligence [58], [59]. For example, *Vata* types may display extroverted but inconsistent social behavior, *Pitta* individuals tend to be direct and leadership-oriented, and *Kapha* types are loyal, steady, and caring. Tools such as NodeXL and IBM Watson [60] Personality Insights can quantify these social patterns and adapt them for Ayurvedic *Prakriti* evaluation.

Similarly, an individual’s way of talking, including tone, pitch, speed, and word choice, can be assessed using NLP, voice analysis software, and sentiment analysis algorithms [61], [62]. These AI models detect communication patterns, emotional undertones, and personality traits, helping to distinguish dosha-specific tendencies: assertive and concise speech in *Pitta*, rapid or scattered speech in *Vata*, and slow, deliberate speech in *Kapha*. ML algorithms continuously refine their predictions by learning from large datasets, enabling assessment of dominance, empathy, or hesitation in communication. Applications like IBM Watson NLP, Google Dialogflow, and Cogito, widely used in voice and text analytics, can be repurposed to provide objective, reproducible insights into an individual’s behavioral constitution and mental attributes (*Sattva, Rajas, Tamas*) for integrative *Prakriti* assessment (Table 5).

**Table 5:** Advanced technologies for behavioral parameters in *Prakriti* assessment

Parameters	Technology used
Friendship tendencies	Social network analysis tools, AI-driven personality profiling, and behavioral analysis algorithms.
Way of talking	Natural Language Processing (NLP), voice analysis software, speech sentiment analysis tools, and AI chatbots.

In summary, analysis of physical and physiological parameters. particularly through imaging systems, wearable sensors, and digital pulse-waveform devices, represents the most developed area of AI-assisted *Prakriti* assessment, supported by established hardware platforms and relatively well-defined analytical pipelines. However, most available studies are based on small or institution-specific datasets and lack external or multicentric validation, which currently limits their clinical generalizability. In contrast, psychological and behavioral AI tools remain largely exploratory [63]. Although emotion-detection, speech analytics, and AI-based cognitive assessment technologies are well established in biomedical and psychological research, their application to *Prakriti* determination is primarily conceptual and requires substantial Ayurveda-specific validation to ensure consistency with classical Ayurvedic constructs.

### 4.2 Overview of multimodal data sources

Assessment of *Prakriti* requires integrating information from multiple domains, including physical, physiological, psychological, and behavioral characteristics. Modern digital technologies, combined with AI and ML algorithms, allow for the systematic collection, quantification, and analysis of these diverse data types (Table 6). By leveraging multimodal data, it becomes possible to create comprehensive, reproducible, and objective profiles of an individual’s constitution, bridging traditional Ayurvedic assessment with contemporary computational approaches.

**Table 6:** Overview of multimodal data sources in *Prakriti* assessment

Domain	Representative parameters	Common digital tools	AI/ML applications
Physical	Height, weight, BMI, skin characteristics, hair type and color, teeth alignment, eye size and color, nail features, palm and lip texture.	Digital surveys, high-resolution imaging, 3D body scanners, spectrophotometers, dermatoscopes	Image analysis, feature extraction, pattern recognition
Physiological	Pulse (Nadi), heart rate, oxygen saturation (SPO_2_), respiration rate, ECG, gait patterns, bowel habits, sleep quality	Wearable sensors (PPG, ECG), accelerometers, smart toilets, sleep monitors, polysomnography	Time-series analysis, signal processing, pattern recognition
Psychological	Cognitive abilities, memory performance, decision-making, emotional regulation	Cognitive testing platforms, EEG devices, voice analysis software, sentiment analysis tools	Predictive modeling, natural language processing, behavioral pattern recognition
Behavioral	Social interaction patterns, communication style, speech characteristics	Social network analytics, NLP tools, voice recognition systems, AI chatbots	Analysis of social behavior, emotion recognition in speech, communication profiling

### 4.3 Barriers to clinical translation of AI-Driven *Prakriti* models

The application of AI-based tools for *Prakriti* assessment faces multiple challenges. A key limitation is the lack of diversity in existing machine learning datasets, which are often developed for populations in high-income countries and may not capture the wide variability of physical and physiological traits in India. Attributes such as hair color, skin pigmentation, nail texture, and body morphology differ significantly across regions and ethnic groups, necessitating data collection from representative populations to ensure accuracy [64], [65]. This limited representation may introduce algorithmic bias and reduce the generalizability of AI-assisted *Prakriti* classification across diverse Indian populations.

Accessibility is another major constraint. Many AI tools rely on wearable devices or continuous monitoring technologies, which are not universally available, and inconsistent usage further reduces their generalizability. Traditional Ayurvedic parameters like *Angula Pramaṇa* (body measurements) and complexion indicators such as *Chaya* and *Prabha* are difficult to quantify using current AI technologies, requiring the development of specialized devices and algorithms. Continuous data collection for sleep, perspiration, or other physiological signals is similarly limited by device availability and user compliance. Behavioral manipulations, intentional or unintentional, may also compromise the accuracy of AI assessments.

Cognitive and psychological assessment platforms, including brain-computer interfaces (BCIs), neuroimaging tools (EEG, fMRI), and mobile apps, face challenges of high cost, technical complexity, signal interference, and standardization issues. Expert interpretation is often required, complicating clinical integration. Social network analysis, NLP, and voice analysis tools also encounter limitations related to cultural biases, accent or emotion misinterpretation, and privacy concerns, while curated or altered social media data may not reflect authentic behaviors.

To address these challenges, future research should focus on creating affordable, culturally adaptable, and standardized AI tools, developing multimodal frameworks that integrate physical, physiological, psychological, and behavioral data, and implementing ethical governance for data privacy and security. Such efforts can enhance the reliability, accuracy, and applicability of AI-assisted *Prakriti* assessment across diverse populations.

## 5 Comparative evaluation of traditional and digital Prakriti assessment approaches

Traditional and contemporary *Prakriti* assessment studies predominantly utilize single-modality approaches, such as questionnaire-based scoring systems or isolated image-based classification. While informative, these methods are limited by subjectivity, low reproducibility, and restricted scalability, reducing their applicability in broader clinical contexts.

**Questionnaire-based assessment:** Multiple questionnaires have been developed to quantify *Prakriti*, varying in length, format (self-assessment or interview), and dosha type coverage (single, dual, or balanced *Prakriti*). However, a significant issue remains the lack of standardization regarding scoring systems, psychometric validation, and instructions for respondents, especially concerning the differentiation between inherent constitution and transient imbalance (*Vikṛti*). Many questionnaires fail to explicitly guide participants to consider lifelong traits rather than recent physiological states, which compromises accuracy. Among validated tools, Ayusoft is a structured software platform for *Prakriti* assessment, employing algorithmic scoring grounded in classical Ayurvedic principles. Peer-reviewed studies report substantial concordance with clinician-based diagnosis, with strong reliability for *Vata* (ICC: 0.72) and *Pitta* (ICC: 0.58), moderate reliability for *Kapha* (ICC: 0.44), and agreement rates up to 80% (κ ≈ 0.7), supporting its role as an objective clinical instrument [13].

**Device-based assessment:** *Nadi Tarangini,* a patented digital pulse diagnosis system, replicates traditional *Nadi Pariksa* using advanced pressure sensors and AI-driven waveform analysis, providing empirical reliability comparable to experienced practitioners [46]. Sensor-based devices for pulse diagnosis adapted from traditional *Nadi Pariksa* principles lack standard protocols, exhibit methodological gaps, and suffer from accessibility issues for independent replication [13].

**Algorithm based assessment:** Additionally, few studies have explored AI-based facial and tongue imaging approaches, where computer vision and machine learning algorithms extract morphological, color, and texture features from high-resolution facial and tongue images to classify *Prakriti* types [20], [35]. Emerging machine learning tools and digital devices for *Prakriti* evaluation show promise but face challenges such as insufficient validation, potential model overfitting, limited public availability of datasets and code, and reliance on subjective physician-derived labels for training data [13].

The CCRAS questionnaire provides a validated, standardized foundation but requires manual administration, whereas AyuSoft offers a more digitally structured, semi-automated workflow with improved reproducibility. AI/ML-based tools take this further by incorporating pattern recognition, biosignal processing, and automated feature extraction, enabling objective analysis beyond subjective questionnaire-based scoring. However, their validation is generally limited to smaller datasets, unlike CCRAS-based tools that have undergone broader field use.

Despite their utility, both CCRAS and AyuSoft rely primarily on self-reported or practitioner-entered data, which may introduce bias. AI/ML-based tools, while promising, often face challenges such as small training datasets, limited external validation, and lack of multicentric, standardized data acquisition protocols. Collectively, these gaps highlight the need for robust multimodal datasets and stronger validation frameworks [66].

## 6 Proposed conceptual framework for AI-based *Prakriti* assessment

This section presents a conceptual and illustrative framework synthesized from existing literature and does not report original experimental data, prototype development, algorithm training, or validation results. The framework is intended to demonstrate how digital inputs described in prior studies may be systematically integrated for future research and should not be interpreted as an operational or clinically validated system.

The framework for AI-based *Prakriti* assessment begins with data collection from a diverse cohort representing major Prakriti types identified by experienced Ayurvedic practitioners. The collected data spans multiple domains: physical attributes via digital questionnaires, high-resolution images of skin, nails, eyes, and anthropometric measurements; physiological parameters through wearable sensors capturing *Nadi Pariksa*, heart rate, respiration, SPO_2_, sleep, and body movements; and psychological and behavioral data including voice recordings for NLP analysis, speech patterns, and social network behavior.

Following collection, data preprocessing and feature extraction standardize formats, handle missing values, and extract features using computer vision models for imaging data, signal processing for physiological signals, and NLP techniques for behavioral inputs. Multimodal data integration then combines these heterogeneous features into unified representations, applying dimensionality reduction techniques such as Principal Component Analysis (PCA) to focus on the most informative attributes.

**Figure 2:**
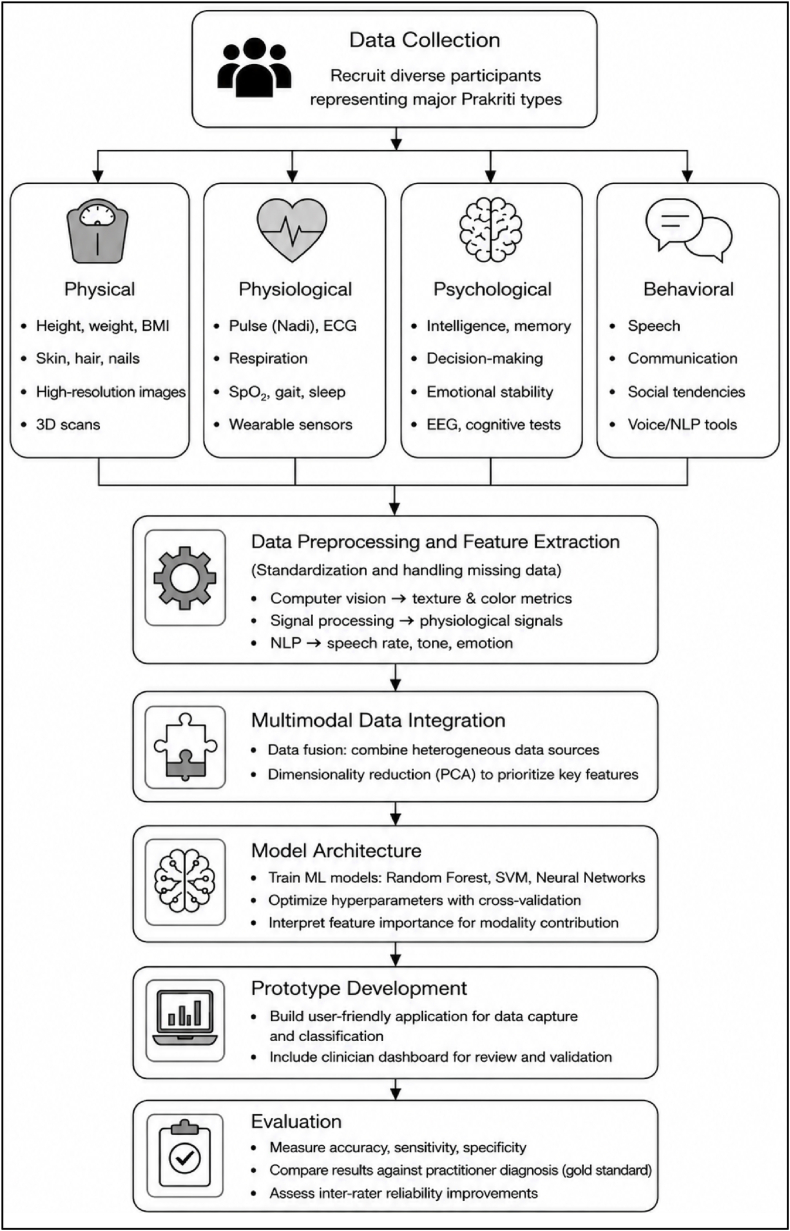
Conceptual framework of Artificial Intelligence enabled *Prakriti* assessment

Next, a supervised ML model, such as Random Forest, Support Vector Machine (SVM), or Neural Networks, is trained on the labeled *Prakriti* data, with hyperparameter optimization performed via cross-validation. Feature importance analyses help interpret the contribution of each modality. A prototype application integrates data capture and AI-driven *Prakriti* classification, complemented by a clinician dashboard for result validation (Fig. 4). Finally, the system is evaluated for accuracy, sensitivity, specificity, and improvements in inter-rater reliability relative to practitioner diagnoses, completing the workflow depicted in Fig. 2.

All figures associated with this section represent illustrative conceptual models only and should not be interpreted as operational prototypes.

## 7 Discussion

### 7.1 Integration of AI into *Prakriti* assessment

The integration of digital technologies and AI into Ayurvedic practice, particularly for *Prakriti* assessment, has enabled more objective and reproducible evaluation of individual constitutions. Traditionally, *Prakriti* assessment relies on practitioner expertise and subjective interpretation of traits such as digestion, sleep patterns, skin type, and behavioral tendencies. Digital questionnaires and AI algorithms, in contrast, allow systematic data capture and analysis, reducing observer bias and improving reproducibility.

AI-based *Prakriti* assessment systems combine data from multiple domains, including physical, physiological, psychological, and behavioral parameters, as summarized in Table 2, Table 3, Table 4, Table 5, Table 6. While basic anthropometric measurements such as height, weight, and BMI are deterministic, machine learning becomes essential when these features are integrated with other physiological and psychological variables for complex classification tasks. For instance, AI algorithms can analyze digestion, skin characteristics, and sleep patterns to identify associations with one or more *doshas*.

The proposed AI platform is a multimodal machine learning system, where structured inputs from questionnaires and sensor-based measurements are processed using supervised learning methods, including logistic regression, random forests, support vector machines, or neural networks. These models are trained on datasets annotated by experienced Ayurvedic practitioners, allowing the system to learn patterns linking specific feature combinations to validated *Prakriti* classifications, including dual-*dosha* (*Samsarga*) and tri-*dosha* (*Sannipata*) types. The role of AI is therefore not to predict temporal changes in *Prakriti*, which is innate, but to provide consistent and objective classification.

**Figure 3:**
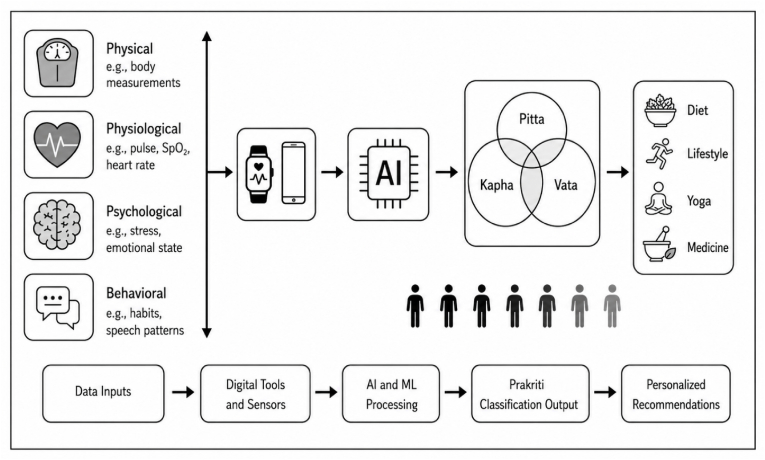
Conceptual workflow of Artificial Intelligence enabled *Prakriti* assessment

AI models in Ayurveda can leverage two complementary data sources: structured knowledge encoded from classical texts into computational ontologies or rule-based frameworks, and annotated clinical datasets curated by expert practitioners. Together, these datasets enable algorithms to recognize characteristic Dosha traits and constitution-linked health predispositions. Once trained, AI systems can provide personalized insights, including diet, lifestyle, yoga practices, and herbal recommendations, which can be updated over time with longitudinal user data (Fig.3). The success of such systems depends on both the robustness and authenticity of training datasets, including demographic diversity, longitudinal information, and expert validation, ensuring that AI outputs remain faithful to classical principles while reflecting real-world variability.

**Figure 4:**
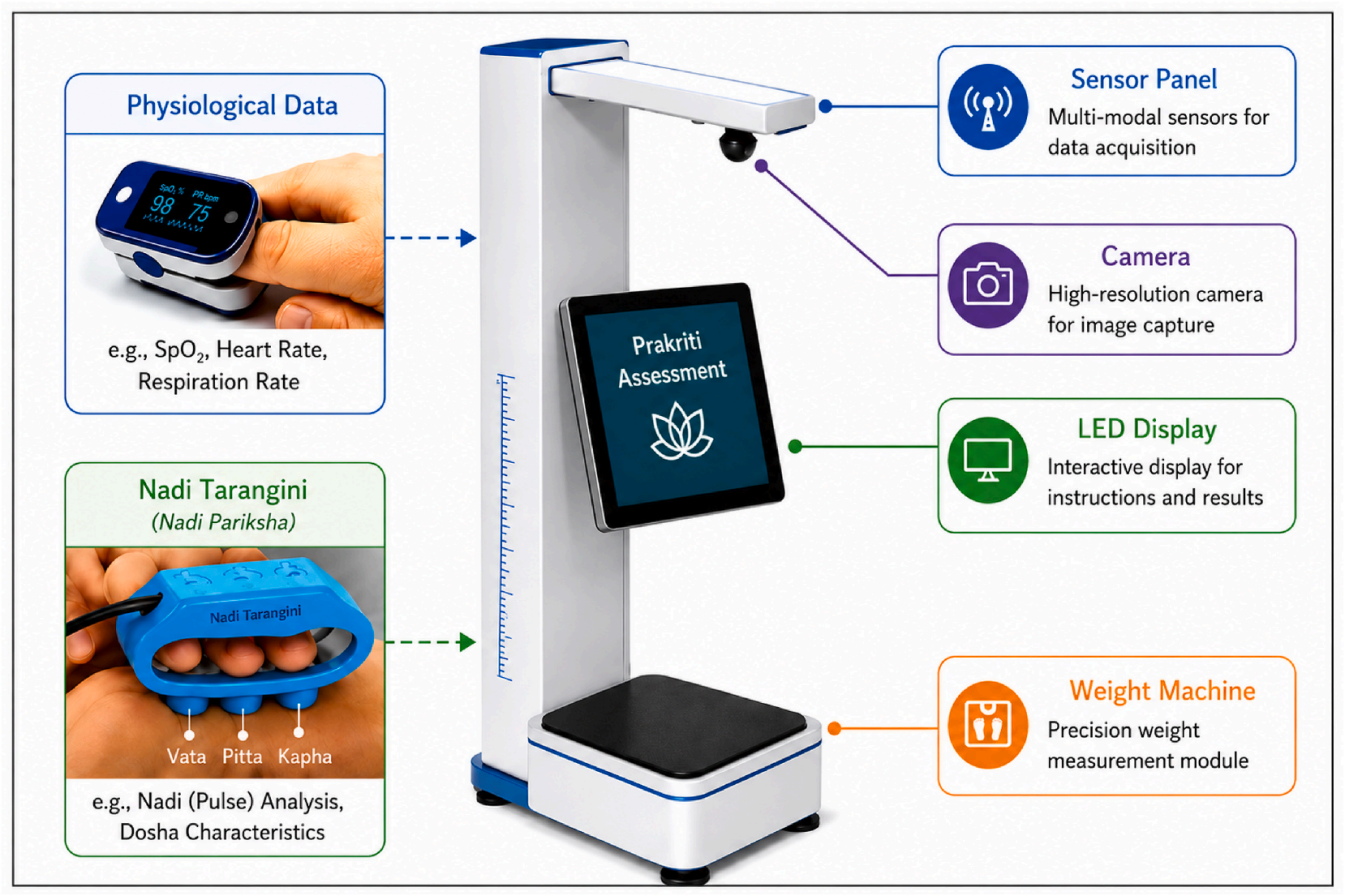
Illustrative conceptual model of Artificial Intelligence enabled *Prakriti* assessment

Instrument-assisted diagnostic tools exemplify AI applications in Ayurveda. The *Nadi Tarangini* system, developed by C-DAC, digitizes pulse waveforms using patented sensors and applies AI algorithms for *dosha* classification, demonstrating reproducible outcomes comparable to experienced practitioners [46]. Such systems are intended to complement, not replace, practitioner expertise, particularly for education, research, and large-scale screening. Similarly, Large Language Models (LLMs) offer potential for mining classical texts and clinical records, enabling automated extraction of *Prakriti*-related rules and constitution-specific patterns [67], [68]. National digital platforms, such as the AYUSH Grid, provide unified repositories of electronic health records, research data, and knowledge bases, facilitating AI-driven innovations in *Prakriti* assessment [69], [70].

### 7.2 Validation and evidence gaps

While several digital technologies demonstrate diagnostic accuracy within biomedical domains, their application to *Prakriti* assessment remains largely inferential. Among currently available tools, structured software-based questionnaires (e.g., Ayusoft) and digital pulse analysis systems (*Nadi Tarangini*) show moderate agreement with clinician-based diagnosis. In contrast, applications involving facial imaging, speech analysis, wearable sensors, and multimodal AI frameworks lack robust validation against Ayurvedic gold standards. These technologies should therefore be regarded as exploratory or supportive rather than definitive diagnostic instruments.

### 7.3 Distinguishing *Prakriti* from *Vikriti* in AI-based assessment

A fundamental methodological challenge in AI-assisted Prakriti assessment lies in distinguishing *Prakriti* (innate constitution) from *Vikriti* (acquired or transient imbalance). *Prakriti* is regarded as stable and determined at conception, whereas parameters such as appetite, bowel habits, sweating, sleep, and emotional states are dynamic and reflect *Vikriti*. Most wearable sensors and digital monitoring tools inherently capture time-dependent physiological data, increasing the risk of conflating transient pathological states with constitutional traits.

Currently, there is a lack of longitudinal studies demonstrating how AI systems can differentiate baseline constitutional signatures from episodic physiological fluctuations. Future AI models must therefore incorporate longitudinal averaging, baseline calibration, and *Vikriti*-flagging algorithms to ensure that digital *Prakriti* assessment remains constitutionally valid and clinically meaningful.

### 7.4 Challenges in implementation

Despite the conceptual promise of AI-assisted *Prakriti* assessment, several implementation challenges persist. A major limitation is the scarcity of large, demographically diverse, and longitudinal datasets representative of Indian populations. Substantial regional and ethnic variability in parameters such as skin pigmentation, hair texture, nail morphology, body frame, and physiological traits is often underrepresented in existing datasets, raising concerns regarding algorithmic bias and limited generalizability.

Additional challenges include insufficient standardization of digital tools, lack of benchmarking against clinician-derived diagnoses, data privacy concerns, and limited interoperability with existing clinical workflows. Multicentric and longitudinal studies involving representative Indian cohorts are essential to enhance reliability, minimize bias, and improve the external validity and clinical applicability of findings. Furthermore, effective implementation necessitates expert-led governance frameworks to standardize assessment instruments, ensure data integrity and input quality, and facilitate iterative system refinement based on structured practitioner feedback.

### 7.5 Economic and feasibility considerations

From a feasibility and pharmacoeconomic perspective, several advanced technologies such as EEG systems, high-resolution 3D body scanners, and continuous biosensing platforms may be impractical for routine deployment in AYUSH primary care settings due to cost, infrastructure requirements, and technical expertise. Scalable *Prakriti* assessment frameworks should therefore prioritize low-cost, smartphone-based tools, simplified sensors, and tiered deployment models aligned with real-world clinical constraints.

### 7.6 Policy recommendations and future directions

Effective and responsible adoption of AI/ML in Ayurveda requires comprehensive policy frameworks under the Ministry of AYUSH. Guidelines should address ethical governance, data privacy, algorithmic transparency, interoperability with clinical workflows, and cultural alignment with Ayurvedic principles [71]. Standardized protocols must guide AI tool development, validation, and deployment, ensuring robust algorithm testing, reproducible outcomes, and user safety. Third-party digital platforms must undergo independent evaluation against clinical benchmarks, including stress testing across diverse demographic and environmental conditions.

Capacity building is essential, focusing on digital health literacy, AI tool interpretation, and responsible usage among Ayurvedic practitioners, educators, and researchers. The Ministry of AYUSH has already initiated IT training programs to support this transition [68]. Furthermore, a central registry of certified AI tools for Ayurveda should be maintained, listing applications that meet criteria for transparency, accuracy, usability, and clinical relevance, ensuring practitioners avoid unverified or pseudoscientific solutions. Adherence to Responsible AI standards, such as those by the Telecommunication Engineering Centre (TEC) and the World Health Organization (WHO), emphasizing fairness, explainability, privacy, and accountability, is critical for safe and evidence-based integration of AI in *Prakriti* assessment and broader Ayurvedic practice [72], [73].

## 8 Conclusion

The integration of Artificial Intelligence into Ayurvedic *Prakriti* assessment represents a promising step toward modernizing and standardizing traditional diagnostic practices. AI-driven tools can enhance accuracy, reproducibility, and efficiency, reducing the subjectivity inherent in conventional evaluations. By aligning the *Tridosha*-based Prakriti framework with contemporary psychological and physiological metrics, such as the Big Five personality traits or Hippocratic temperaments, a more evidence-based and globally relevant understanding of individual constitution can emerge. This convergence of ancient wisdom and modern technology not only strengthens the scientific credibility of Ayurvedic practice but also opens avenues for personalized healthcare, preventive strategies, and broader acceptance of Ayurveda in the global health landscape.

## Declaration of generative AI in scientific writing

We hereby declare that no generative AI tools were used in the drafting, writing, or editing of this manuscript. All writing and editing were performed by the authors.

## Author contributions

ARK: Conception, conceptualizing, original draft preparation, reviewing and editing, BK:

Supervision and writing, SS: Reviewing and editing, SGS: Reviewing, SGR: Original draft

preparation, data curation, and pictures

## Funding sources

This article did not receive any funds.

## Declaration of Competing Interest

Nil

## List of Abbreviations

AI: Artificial Intelligence
ML: Machine Learning
BMI: Body Mass Index
NLP: Natural Language Processing
EEG: Electroencephalography
ECG: Electrocardiography
fMRI: functional Magnetic Resonance Imaging
PPG: Photoplethysmography
SVM: Support Vector Machine
PCA: Principal Component Analysis
CCRAS: Central Council for Research in Ayurvedic Sciences
AYUSH: Ayurveda, Yoga & Naturopathy, Unani, Siddha, Sowa-Rigpa and Homoeopathy
